# Antidote or Poison: A Case of Anaphylactic Shock After Intra-Articular Corticosteroid Injection

**DOI:** 10.7759/cureus.1625

**Published:** 2017-08-29

**Authors:** Pooja Sethi, Jennifer Treece, Chidinma Onweni, Vandana Pai

**Affiliations:** 1 Department of Cardiology, Quillen College of Medicine, East Tennessee State University; 2 Internal Medicine, Quillen College of Medicine, East Tennessee State University

**Keywords:** glucocorticoid, anaphylactic shock, ige-mediated hypersensitivity reaction, corticosteroids, intra-articular corticosteroid injection

## Abstract

Although glucocorticoids are often used as an adjunct to epinephrine to treat anaphylactic shock, glucocorticoids can also be a rare cause of anaphylactic shock. Only through the administration of a challenge dose of different glucocorticoids and different substrates that glucocorticoids are delivered in can the determination be made about which glucocorticoid or accompanying solvent may be the culprit which caused the anaphylactic reaction. These challenge tests should only be performed in a controlled environment as repeat anaphylaxis is a risk, especially if the patient has a history of glucocorticoid-induced anaphylaxis.

## Introduction

Anaphylactic shock is a rapid, potentially life-threatening condition that is caused by a severe allergic reaction to an allergen. It is treated with emergent intramuscular (IM) epinephrine [1]. Glucocorticoids may also be used in the treatment of anaphylactic shock following epinephrine administration. Glucocorticoids are a very rare cause of anaphylactic shock. Hypersensitivity and anaphylaxis exist in a continuum, anaphylaxis being the most severe form. Hypersensitivity reactions secondary to glucocorticoids are not uncommon. Anaphylaxis secondary to glucocorticoids, however, is uncommon with a reported incidence of less than 0.1% of all parenteral administrations of glucocorticoids [2]. Still more uncommon is anaphylaxis secondary to an intra-articular injection of a glucocorticoid, as is presented in this case.

## Case presentation

A 52-year-old woman presented to her rheumatologist’s office for an intra-articular betamethasone injection to relieve pain from osteoarthritis, which was the second intra-articular betamethasone injection that she had ever received with the previous injection without incident. She denied any known allergies to food, medications, or latex. Within minutes of the intra-articular injection, she developed nasal pruritus and conjunctival injection and had to lie down due to acute onset of dizziness. Simultaneously, she also developed dyspnea associated with angioedema and a generalized urticarial rash. On physical exam, she was hypotensive with blood pressure of 70/50 mmHg, tachycardic with a pulse of 130 beats per minute, and hypoxic with oxygen saturation decreasing to 70% on room air. Emergency medical services (EMS) was called, and the patient was urgently transferred to our hospital. She required intubation due to hypoxia and severe respiratory distress. She was given two doses of 0.3 mg (concentration of 1:1000) IM epinephrine injection to treat anaphylactic shock. She was also given intravenous (IV) ranitidine, diphenhydramine, and was started on a saline bolus. IV steroids were avoided since the anaphylactic reaction was thought to be caused by her recent betamethasone injection. With treatment, her blood pressure improved and her generalized edema resolved. She was successfully extubated the next day and was discharged home without any sequelae.

Six weeks later after informed consent was obtained, intradermal skin testing was performed. A strong allergic reaction to betamethasone was elicited on the intradermal skin test as well as a moderate reaction to prednisone. Tests for methylprednisolone, dexamethasone, triamcinolone, lidocaine, latex, and an injected suspension including polyethylene glycol (macrogol), polysorbate 80, mono and dibasic sodium phosphate were all negative. It was thereby determined that the patient’s anaphylactic reaction was secondary to betamethasone itself and not due to the injected suspension.

## Discussion

Risk factors

A history of allergic reaction, particularly a history of anaphylaxis, predisposes patients to future anaphylactic reactions. Patients with asthma tend to have an allergic component that is exacerbated when exposed to a trigger. Less than 2% of cases of anaphylaxis become fatal [3], and approximately 80-87% of the fatal cases of anaphylaxis are secondary to delayed intramuscular epinephrine injection [4].

Clinical features

Anaphylaxis is characterized by rapidly progressive cardiopulmonary compromise with acute respiratory failure and hypotension following exposure to a trigger [5]. Signs and symptoms of anaphylaxis develop within 30 minutes of exposure to an allergen [5]. Over 90% of patients with anaphylaxis develop cutaneous manifestations, including angioedema, urticaria, pruritus, or flushing [6]. In addition to cutaneous manifestations, patients with anaphylaxis can also develop laryngeal edema with inability to protect their airway, bronchospasm, and hypotension [5].

Diagnosis

Anaphylaxis may occur secondary to medications, insect stings, or foods [1]. Anaphylactic reactions occur rapidly following exposure to the allergen, so determining the etiology of the anaphylactic reaction often requires discovering the exposure history. Measurement of serum tryptase levels confirms the diagnosis of anaphylaxis as the serum tryptase level will be elevated in a patient in anaphylactic shock, peaking one to two hours following anaphylaxis, and remaining elevated for several hours following the onset of anaphylaxis [1,5]. Use of a challenge test can be performed by administering small doses of multiple potential allergens to a patient to determine which allergens the patient is allergic.

Treatment

Emergent administration of epinephrine intramuscularly (IM) is the primary treatment of anaphylactic shock [1,5]. Secondary treatments that can be given in addition to epinephrine to treat anaphylaxis include antihistamines, steroids, and beta-agonists [5]. Avoidance of the allergen that induced the anaphylactic reaction is the mainstay of preventing future anaphylactic reactions. All patients with a history of anaphylactic reaction need to be aware of what triggers their anaphylactic reaction and need to carry epinephrine with them as an emergency medication. Epinephrine IM may be either self-administered or administered by a bystander if the patient were to come into contact with the allergen in the future and develop a subsequent anaphylactic reaction [6].

In the cases where treatment with corticosteroids may be required in the future, therapeutic options for patients with documented steroid allergy include the use of alternative steroid preparations, desensitization, and, possibly, the use of monoclonal anti-IgE antibodies.

Summary

Although systemic glucocorticoids are frequently used to treat allergic reactions, including anaphylaxis, there are rare case reports of hypersensitivity to systemic corticosteroids. However, cases of anaphylactic shock after intra-articular injection of steroids are exceedingly rare. There are case reports of anaphylaxis secondary to polyethylene glycol (macrogol), a latex-like substance that is used as a solvent to suspend solutions like corticosteroid injections [7-8]. The patient in this case was tested for allergic reaction to polyethylene glycol (macrogol) and was found to not be allergic to this substance; her anaphylactic reaction was to the betamethasone itself and not to the accompanying solvent. Her initial intra-articular joint injection did not elicit an allergic response likely due to her body having not been exposed to betamethasone before and therefore not ready to mount an allergic reaction. When she was exposed to betamethasone again during her second intra-articular injection, she was sensitized to betamethasone from her previous exposure and therefore ready to mount a response.

The exact pathophysiological mechanism of anaphylaxis secondary to corticosteroid has not been elucidated, but it is thought to be an immediate IgE-mediated hypersensitivity reaction [9]. Skin prick testing of glucocorticoids should be done in controlled settings because patients sensitized to one group of glucocorticoids may not exhibit cross-reactivity to all types of glucocorticoids. Careful challenging of patients in controlled settings helps identify safe glucocorticoids that do not cause anaphylaxis and therefore can be used to treat any future allergic or anaphylactic reactions. There is potential that oral macrogol may be used in challenging patients following anaphylaxis after intra-articular corticosteroid injection, but it would not have been useful for the patient presented in this case as she was not allergic to macrogol but to betamethasone [10].

## Conclusions

Because anaphylaxis secondary to corticosteroids is, albeit a rare, but potential reaction to intra-articular joint injections, epinephrine should always be kept in rheumatology offices as timely injection of epinephrine may have alleviated the need for intubation in the patient presented in this case and can be lifesaving.
